# The association between sleep problems and academic performance in primary school-aged children: Findings from a Norwegian longitudinal population-based study

**DOI:** 10.1371/journal.pone.0224139

**Published:** 2019-11-07

**Authors:** Kjell Morten Stormark, Hedvik Elisabeth Fosse, Ståle Pallesen, Mari Hysing

**Affiliations:** 1 Regional Centre for Child and Youth Mental Health and Child Welfare, NORCE Norwegian Research Centre, Bergen, Norway; 2 Department of Health Promotion and Development, University of Bergen, Bergen, Norway; 3 Norwegian Competence Centre for Sleep Disorders, Haukeland University Hospital, Bergen, Norway; 4 Department of Psychosocial Science, University of Bergen, Bergen, Norway; Hamamatsu University School of Medicine, JAPAN

## Abstract

The purpose of this study was to examine the longitudinal association between concurrent, transitory and persistent difficulties initiating and maintaining sleep (DIMS) on academic performance in children in a. longitudinal child-cohort (N = 3986) targeting school-aged children when they were 7–9 years (T1) and 11–13 years (T2) old, whilst controlling for mental health problems. DIMS were parent-reported at T1 and T2 and academic performance teacher-reported at T2. Mental health was based on child self-report at T2 using the Strength and Difficulties Questionnaire (SDQ). In all, 10.6% (n = 423) of the children had poor school performance at T2. These had more symptoms of externalizing and internalizing mental health problems (p. < 001) compared to their peers at T2. A logistic regression analysis showed that both concurrent DIMS (at T2, but not at T1) and persistent DIMS (at both T1 and T2) was associated with elevated risk of poor academic performance. After controlling for mental health problems, only persistent DIMS was associated with increased risk of poor academic performance. Transitory DIMS (DIMS at T1 but not at T2) did not increase the risk of later poor academic performance. A mediation analysis also revealed that the association between DIMS and poor school performance was mediated by mental health problems, in addition to an overall significant direct relative effect of DIMS on poor school performance in the persistent DIMS group. These findings support the notion that sleep problems in children are associated with impaired academic performance, and extends past findings demonstrating that sleep problems may not increase the risk of poor academic performance unless they persist over time. The negative effects of persistent sleep problems suggest that more emphasis should be put on preventive interventions of sleep problems in school-aged children.

## Introduction

Sleep problems are common in children [1] and adolescents [2–7],with estimated prevalence ranging from 11% [8] to 47% [9] depending on factors like study sample, mode of assessment [10] and self- [11] vs caregiver [12]-report, and how sleep problems are defined and conceptualized [13]. A distinction is often drawn between sleep problems due to symptoms of parasomnia (dissociated sleep states such as in sleep walking) or dyssomnias (difficulties initiating and/or maintaining sleep, such as in insomnia[14]], of which the latter set of symptoms appears to most frequent in children [15]. While it previously was assumed that children outgrow these problems [10, 15, 16], recent studies on sleep problem trajectories show that there actually is an increase during late childhood [17]. Prevalence estimates based on cross-sectional community surveys suggests that one out of five pre-adolescent children [18] and adolescents [19] have symptoms of insomnia. Children, who according to their parents have persistent symptoms of difficulties falling asleep and/or nocturnal awakenings also have twice the risk of fulfilling the Diagnostic and Statistical Manual of Mental Disorders (DSM-5; [20] criteria for insomnia in late adolescence [21].

Sleep plays an important role in fostering both short- and long-term developmental regulation of cognition, emotions and behaviour [22–24], as proper restitution is necessary for optimal neurocognitive and emotional functioning [25]. In particular, the association with neurocognitive functions involving the prefrontal cortex during childhood and early adolescence has attracted increased attention [26] as this constitutes a period of important neural maturation and brain growth and reorganization [27, 28]. Subjective self- or parent-report of children’s difficulties initiating and maintaining sleep have, in addition to objective actigraphy or polysomnography recordings during sleep, also served as indices for sleep quality [29], which have been found to be more strongly related to mental well-being [30] and academic performance in young adults [31] and children [32] than measures of sleep duration. The concept of sleep quality implies that good sleep is also subjective, which objective measures of sleep cannot always assess [33, 34].

In a number of studies, measures of poor sleep quality has been found to be related to degraded performance on a number of cognitive tasks, particularly complex tasks involving attention [35] and memory [36] and continuous performance [37] and tasks drawing on executive functions and global intelligence measures [38]. The results from a meta-analytic review [29] also suggested that sleep quality have a stronger, statistically significant yet modest, relation to school performance in children than do sleep duration. Larger effects sizes were obtained in studies targeting children and drawing on subjective sleep assessment methods than studies targeting adolescents and drawing on objective sleep assessment methods, even though there was considerable variability in the effect sizes between the reviewed studies, among other due to age and gender effects. Results from a validation study on children’s self-reported sleep functioning [39], and a cross-lagged panel study [40], not covered in the meta-analysis, also found sleep quality to be related to teacher-reported and self-ratings of academic affiliation, in the latter study primarily in boys [40].

The study of the association between sleep and academic performance in children leans itself primarily on findings from cross-sectional studies [29]. The few existing longitudinal studies have primarily examined the association with sleep duration [41–45].The purpose of the present study was to examine the longitudinal association between parent-report of children’s difficulties initiating and maintaining sleep ((DIMS) at two time-points, in late childhood (T1) and early adolescence (T2) on school performance, drawing on data from the Bergen Child Study (BCS). The BCS is a multi-wave child cohort study of mental health targeting three age cohorts of children in the Bergen municipality, Norway. As this association may be temporary [15] or chronic [46], we were interesting in distinguishing between the associations between concurrent, transitory or persistent DIMS on academic performance in primary school children, whilst controlling for gender [47] pubertal status [48], socio-economic status [49, 50] and symptoms of mental health problems [51], which is found to influence both sleep [52] and academic performance [53]. In order to determine the effects of mental health problems on the association between DIMS and academic performance, a mediation analysis was conducted.

## Materials and methods

### Participants

The current sample consisted of children taking part in the first two waves (T1-T2) of the BCS, carried out in the autumn of 2002 and the spring of 2006, respectively. The BCS is a longitudinal prospective study of children’s mental health from primary academic age to adolescence in the municipality of Bergen, Norway (http://uni.no/en/bergen-child-study). The protocol and the population of the BCS have been described in detail in separate pervious publications [54, 55].

The present study draws on data obtained from parents at T1 and from parents, teachers and children at T2. In all, 9430 school-aged children were targeted at T1 when they were 7–9 year olds. Parents of 7007 children consented to their child’s participation in the BCS. At T2 (when the children were 11–13 year olds), parents of 5661 children consented to their child’s participation, comprising a longitudinal sample of 4028 children who participated at both T1 and T2. The longitudinal sample did not differ from the sample that was lost to follow-up on their DIMS scores at T1, according to results from the Kruskal-Wallis test, with mean DIMS scores of 0.09 and 0.11 for the longitudinal sample and the sample lost to follow-up, respectively. Complete information was available for 3480 children. An additional 27 children were excluded from the analysis due to parent-reported intellectual disability. Thus, the total current study population comprised 3453 children (47.2% boys), which constituted 35.1% of the original target population, and 49.3% and 61.0% of the study population at T1 and T2, respectively. The current study population did also not differ on the DIMS T1 scores, neither from the sample lost to follow-up (K-W test (df = 1) = 0.19) nor the sample that had been excluded due to missing data or parent-reported intellectual disability (K-W test (df = 1) = 0.14). The mean DIMS scores for the current study sample and the sample excluded from the analyses were 0.09 and 0.11, respectively.

### Instruments

Sleep problems were assessed with one question encompassing difficulties with initiating and/or maintaining sleep through parental ratings at both T1 and T2 based on the question; “Does the child have problems initiating or maintaining sleep (DIMS)?” This was rated on a three-point (not agree, partly agree, agree) scale. All children whose parents agreed or partly agreed to the question were defined as having problems with falling asleep and/or with nocturnal awakenings. This operationalization has previously been successfully applied in papers from the BCS [21, 56]. Concurrent sleep problems were defined as having DIMS at T2, but not on T1. Transitory sleep problems were defined as having DIMS at T1, but not on T2. Persistent sleep problems were defined as having DIMS at both T1 and T2.

At T2 the teachers indexed the children’s academic performance by answering the question: “How do you evaluate the child’s academic performance?” on a four-point scale (very high, high/average, low and very low). All children whose teacher rated their academic performance to be “very low” or “low” constituted the poor academic performance group. This group was contrasted with the rest of the children who constituted an average-to-good academic performance group, based on their teacher’s ratings.

The children’s mental health was assessed by the children’s self-report on the Strengths and Difficulties Questionnaire (SDQ) at T2 [57, 58]. The SDQ is a screening questionnaire for children aged 4–16 years, comprising 25 items describing positive and negative attributes of children distributed across five subscales, each with five items: (1) emotional symptoms, (2) conduct problems, (3) hyperactivity-inattention problems, (4) peer relationship problem, and (5) pro-social behaviour. Each item is scored on a three-point scale (“not true”, “somewhat true”, and “certainly true”). In addition, the “emotional symptom subscale”and the “peer problems subscale” are found both conceptually and statistically to comprise a broader internalizing subscale, while the items on the two subscales “conduct problems” and “hyperactivity-inattention problems” can be subsumed into an externalizing subscale [59]. Since the purpose of the present study was to determine the relationship between predictor and outcome variables from an epidemiological study, and not to screen for disorder, we used the broader internalizing and externalizing subscales here. Familial socioeconomic status (SES) was indexed by economy and perceived economic well-being on a five point scale from “very good” to “very poor”. The child’s level of pubertal status at T2 was indexed by parent-reports on a single question “In puberty, the body changes from being a child into being an adult. In some children, the changes start early and, in other children, the changes start later. Compared with other children of the same age, did the pubertal development start earlier, at the same time or later?” This was scored on a five-point scale (“much earlier”, “earlier”, “at the same time”, “later” and”much later”.

### Statistical analyses

We conducted Pearson chi-square tests and Kruskal–Wallis analyses for the independent samples to examine differences in demographics and self-reported mental health between children with and without poor academic performance. Non-parametric tests were chosen due to non-normality of the data. A logistic regression analysis was used to explore the association between DIMS and academic performance. Both unadjusted/crude (analyzing the bivariate relationships between the single possible confounding variables and academic performance) and analyses where all potential explanatory variables were entered simultaneously (adjusted) were conducted. The confounders we adjusted for were: Gender, Pubertal status, SES, Internalizing and Externalizing mental health problems. Finally, we conducted a fully adjusted analysis controlling for all potential confounders. Results are presented as odds ratios (OR) with 95% confidence intervals (95% CI). The mediating effects of Internalizing problems and Externalizing problems on the relationship between DIMS and poor school performance was estimated based on the Ordinary Least Squares Linear Regression model in the PROCESS macro for SPSS [60] yielding, based on 5,000 bootstrap samples, a logistic regression path analysis of multiple parallel mediation effects model for estimation of the relative direct and indirect effects of the T2 variables on the relationships between the multi-categorical antecedent [61] DIMS groups (with the no DIMS group as the reference group) and the dichotomized outcome variable Poor school performance.

All analyses were carried out using the statistical software package (SPSS) version 24 (IBM Corp, IBM SPSS Statistics for Windows, Version 24.0, Armonk, NY, USA, IBM Corp).

### Ethics

The National Data Inspectorate and the Regional Committee for Medical and Health Related Research Ethics in Western Norway have approved both waves of the BCS (REK 184–01 and REK 062–06). Parents provided their informed consent for participation at both T1 and T2.

## Results and discussion

At T1 a total of 270 children (7.8% of total study population) were reported by their parents to have difficulties initiating and maintaining sleep (DIMS), of which 132 children also had DIMS at T2. Almost one-half of the children with DIMS at T1 also had DIMS at T2, and hence constituted the Persistent DIMS group. The remaining 138 children who had DIMS at T1 but not at T2 comprised the Transitory DIMS group. An additional 265 children (7.7% of the study cohort) had DIMS at T2 but not at T1 constituted the Concurrent DIMS group. The rest of the children (n = 2772, 80.3%), who were not reported to have sleep problems at any time, comprised the No DIMS comparison group.

A total of 362 children (10.5% of the current study population) were reported by their teacher to evidence poor academic performance. Comparisons between the poor academic performance and control group constituting the children with average-to-good academic performance revealed statistically significant differences in terms of DIMS, pubertal development, family economy, and level of parental education and mental health scores (see Table 1). The low academic performance group comprised a significant larger proportion of children with DIMS, larger proportion of children with delayed pubertal development, less affluent family economy, lower levels of parental education and more mental health problems than the control group (all comparisons p < .001).

**Table 1:** Sample characteristics. Differences in the distribution of DIMS, pubertal status, family economy and mental health scores between children with poor academic performance and children with average-to-good academic performance.

	Poor academic performance	Average to good academic performance	Statistical significance
	*% children at T2*	*% children at T2*	**χ *p***
**DIMS**			46.96 < .001
-No DIMS	74.7	84.9	
-Transitory DIMS	2.6	4.4	
-Concurrent DIMS	13.4	7.4	
-Persistent DIMS	9.3	3.4	
**Pubertal status**			13.64 < .001
-Early	7.2	16.1	
-Average	76.5	75.8	
-Late	12.8	8.1	
**Family economy**			23.66 < .001
-Good	62.5	72.7	
-Average	33.1	25.7	
-Poor	4.4	1.6	
	*Means (SE*	*Means (SE*	***K-W test p***
**Mental health**			
-SDQ Internalizing problems	4.11(0.18)	2.40(0.44)	106.03 < .001
-SDQ Externalizing problems	5.33(0.16)	3.18(2.64)	164.18 < .001

*Note* DIMS = Difficulties initiating and maintaining sleep, K-W test = Kruskal-Wallis test

The results from the crude logistic regression analysis showed that children with transitory DIMS did not have increased risk for poor academic performance at T2, compared to the no-DIMS children (OR 0.69; 95% CI 0.35–1.37) or in the models adjusting for gender, pubertal status and SES. The models additionally adjusting for mental health problems yielded an almost significantly lower risk of poor academic performance in the transitory DIMS compared to the no-DIMS group (Fully adjusted model: OR 0.50; 95% CI 0.25–1.02; see Table 2).

**Table 2:** Sleep problems and school performance. Logistic regression models analyzing the relationship between sleep problems and poor school performance at T2 in children transitory, concurrent or persistent DIMS (Difficulties initiating and maintaining sleep; N = 3543).

	Transitory (only at T1)		Concurrent (only at T2)		Persistent (both at T1 and T2)	
	OR(95% CI)	*p*	(95% CI)	*p*	(95% CI)	*P*
Model A: DIMS	0.69(0.35–1.36)	.28	2.12(1.51–2.98)	< .001	3.15(2.07–4.78)	< .001
Model B: Model A+Gender	0.69(0.35–1.37)	.28	2.11(1.50–2.97)	< .001	3.09(2.03–4.70)	< .001
Model C: Model B+Pubertal status	0.69(0.35–1.38)	.29	2.05(1.45–2.89)	< .001	3.04(1.99–4.63)	< .001
Model D: Model C+SES (Perceived family economy)	0.64(0.32–1.28)	.21	1.97(1.40–2.70)	< .001	2.78(1.81–4.25)	< .001
Model E: Model D+ SDQ Internalizing problems	0.51(0.25–1.04)	.06	1.37(0.95–1.98)	.09	2.05(1.32–3.20)	< .01
Model F: Model D+ SDQ Externalizing problems	0.55(0.27–1.12)	.10	1.36(0.95–1.96)	.10	2.07(1.32–3.25)	< .01
Model F: Fully adjusted model (Model A+B+C+D	0.50(0.25–1.02)	.06	1.19(0.82–1.73)	.35	1.87(1.19–2.95)	< .05

*Note* SDQ = Strengths and Difficulties Questionnaire

Children with concurrent DIMS were in the crude model found to have an increased risk of poor academic performance at T2 compared to no-DIMS children (OR 2.11; 95% CI 1.50–2.97), but in the fully adjusted model, this association did not longer remain significant (OR 1.19; 95% CI 0.82–1.73).

Finally, persistent DIMS was a significant predictor for low academic performance, both in the crude (OR 3.09; 95% CI 2.03–4.70) as well as in the fully adjusted model (OR 1.87; 95% CI 1.19–2.95).

**Fig 1:**
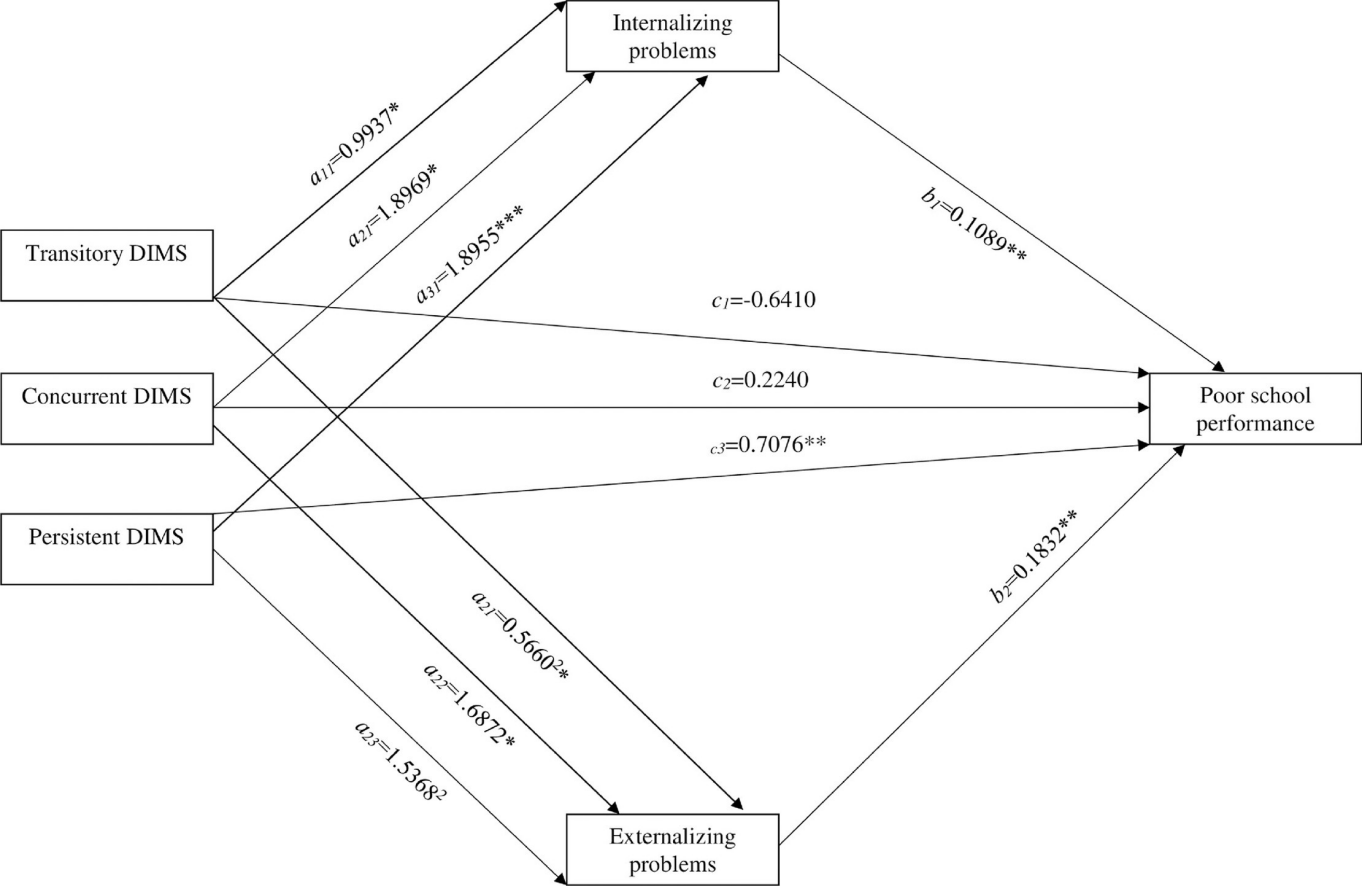
Parallel multiple mediator model between antecedent DIMS and school performance. Fig 1 represents a graphic depiction of the odds ratio reflecting in a parallel multiple mediator model with two mediators (Internalizing problems and externalizing problems) between the multi-categorical antecedent DIMS groups (with the No DIMS-group as the reference) and Poor school performance. The _*a11-13*_paths depict the relative direct effects in terms of Ordinary Least Squares Linear (OLS) regression coefficients of the Transitory DIMS (_*a11*_), the Concurrent DIMS (_*a21*_) and the Persistent (_*a31*_) DIMS groups (with the No DIMS group as reference) on the mediators Internalizing problems. The _*a21-23*_ paths depict the relative direct effects in terms of OLS regression coefficients of the Transitory DIMS (_*a21*_), the Concurrent DIMS (_*a22*_) and the Persistent (_*a32*_) DIMS groups (with the No DIMS group as reference) on the mediator Externalizing problems.The _*b1-2*_ paths depict the relative (to the No DIMS group) indirect effects in terms of OLS regression coefficients reflecting the three relative paths for the effect of DIMS on poor school performance through DIMS’ effect multiplied by the three groups (relative to the No DIMS group as reference through DIMS’ effect on Internalizing (_*b1*_) and Externalizing (_*b2*_) problems. The _*c1-3*_ paths reflecting the relative direct effects in terms of OLS regression coefficients for the Transitory DIMS (_*c1*_*)*, the Concurrent DIMS (_*c2*_*)* and the Persistent DIMS (_*c3*_*)* groups (with the No DIMS group as reference).

The results from the path mediation analysis revealed first, as shown in Fig 1, that there were mediation effects between DIMS and poor school performance from both internalizing and externalizing mental health problems for the Transitory DIMS, Concurrent DIMS and the Persistent DIMS groups (all relative to the No DIMS group; all comparisons p < .05). Controlling for these mediation effects in parallel, there was still an overall significant direct relative effect of DIMS on poor school performance (χ(3) = 14,26 p < .001), due to a significant relative direct effect in Persistent DIMS group (log odds ratio of 0.7076, p < .001), while not in the Concurrent DIMS group (log odds ratio of 0.2240, p = .23) or the Transitory DIMS group (log odds ratio of -0.6410, p = .08). We also tested for relative direct effects when controlling for Pubertal status and Perceived Family economy in a parallel mediation model, which also evidenced a significant relative direct effect in Persistent DIMS group (log odds ratio of 0.6336, p < .001), but not in the Concurrent DIMS group (log odds ratio of 0.1755, p = .35) or in the Transitory DIMS group (log odds ratio of -0.6863, p = .06), even though the relative direct effect in the Transitory group approached statistical significance.

The main finding in the present study was that parents’ reports of their children’s sleep problems in terms of difficulties initiating and maintaining sleep when they were 7–9 year olds was associated with the children’s teacher-reported school performance three years later. After controlling for the confounding effects of demographic and in particular internalizing and externalizing mental health problems, sleep problems were still associated with an increased risk of poor school performance, in the Persistent DIMS group. This association was partly mediated by the children’s self-reported internalizing and externalizing mental health problems. Neither the children in the Concurrent nor in the Transitory DIMS groups had any increased risk of poor school performance, after controlling for mental health problems. Finally there was no direct effect of sleep problems on school performance in these two groups, suggesting that the association between sleep problems and poor school performance was mediated by mental health problems.

Children in the Persistent DIMS group had increased risk of poor school performance adjusting for the demographic and mental health factors. Neither children in the Concurrent nor in the Transitory DIMS groups had any increased risk of poor school performance compared to children in the No DIMS group, after adjusting for the demographic and mental health factors. In the transitory DIMS group, sleep problems at T1 were, after controlling for the effects of the demographic and mental health factors, associated with a reduced risk of poor school performance, although not significantly so.

The finding that persistent sleep problems increased the risk of poor school performance accords with both results from the meta-analytic review of cross-sectional findings [29, 62], cross-sectional findings from the BCS on sleep and school absence in adolescents [63] and long-term findings from the Dunedin birth cohort that persistent sleep problems during childhood predict impaired cognitive function in adolescence [64], even though problems initiating and maintaining sleep in these studies have been assessed by more than a single question used in the current study

The association between sleep problems and school performance was mediated by the children’s self-reported level of internalizing and externalizing mental health problems. This is in line with previous findings of strong bidirectional associations both between sleep and mental health [13, 65], and between school performance and mental health [66, 67] in children.

There was no cross-sectional association between sleep problems and school performance in the children in the Concurrent DIMS group, after controlling for confounders and the association was fully mediated by symptoms of mental health problems. The mediation through children’s self-reported level of internalizing and externalizing problems suggests that sleep problems may exert its influence on school performance through symptoms of mental health problems during childhood [68], possibly through neuropsychological and psychosocial daytime functioning [69], emotional [52], behavioral [70] regulation and executive functions [71].

In contrast to children in the Concurrent DIMS group there was a direct effect of sleep problems on school performance in the children in the Persistent DIMS group. This could be attributed to the magnitude of the sleep problems, as it is likely that children with persistent problems also are more affected by them. This is also in line with our previous findings that children in this cohort with persistent DIMS have twice the risk of fulfilling the DSM-5 criteria for insomnia when they reach late adolescence [21]. It could also be that that sleep problems in this group reflects comorbid conditions such as chronic illness [72], neurodevelopmental disorders [73] or psychosocial stressors [74, 75], which affects sleep efficiency and architecture.

In the transitory DIMS group, after controlling for self-reported internalizing and externalizing problems, former sleep problems actually emerged as a possible protective factor against poor school performance (see first column in Table 2). Even though the association did not reach statistically significant levels, one could speculate if remitting sleep problems in these children could be due to positive changes in their environment which also improved their school performance. As an example sleep hygiene practices such as maintaining a consistent bedtime routine and sleep schedule have been found to not only improve sleep [76] but also academic performance [77] and mental health [78] in children and adolescents.

The limitations of the current study should be noted. First, both sleep and academic performance were measured using single items, not targeting the nature of neither the sleep nor the academic performance problems in any depth. Second, DIMS was assessed by parent-report alone. Even though many studies find that parental reliably identifies children’s sleep problems [79], it is also shown that they could be prone to underestimate certain difficulties such as problems falling asleep and nocturnal awakenings, particularly in older children [4]. Third, as we have no information about neither the child’s academic performance, mental health problems nor any of the mediating factors at T1 we could not control for these in the analyses. Self-report on the SDQ was not available for the age range of the study sample at T1. Another important limitation was that we did not obtain any measure of sleep duration, and thus cannot determine the extent to which this variable overlap with sleep quality [80], and test the direct and indirect [81] effects on school performance [82]. Finally, unmeasured and potentially confounding factors could account for the association between sleep and academic performance for the group of children with persistent DIMS. They may have other characteristics such as neurological conditions or chronic disorders that both are risk factors for sleep problems in general as well as poor academic performance [83].

The main strengths of the present study were the large sample size, the longitudinal design and the multi-informant design. The present study is one of only few longitudinal prospective studies on sleep and academic performance in unselected child cohorts. As we used parent reported information on DIMS, self-reported mental health problems and teacher-report on academic performance, the risk that the results were due to common method bias [62], which has been pointed out as a limitation in the past studies on adolescents’ sleep and school performance [84], is substantially reduced.

## Conclusions

Taken together, these findings add to the current knowledge about the relationship between sleep problems and academic performance by examining this relationship longitudinally, and at the same time controlling for mental health problems, gender, pubertal status and socioeconomic status. We found that concurrent sleep problem, in terms of children’s difficulties initiating and maintaining sleep at age 10–12 years did not increase the risk for poor academic performance after controlling for mental health problems. However, if the sleep problems persisted from age 7–9 until age 10–12, it increased the risk for poor academic performance, even after controlling for mental health problems. These findings emphasize the importance of assessing sleep problems in young children. While children’s sleep problems often are overlooked [85], these problems are often also associated with a cluster of identifiable medical history [51], familial [75], and psychological [13] risk factors. This suggests that early intervention is possible, also in order to prevent adverse effects on later academic performance, in accordance with what the results from trials on school-based sleep education programs for children have shown [86]. Future studies are needed in order to disentangle the exact mechanisms behind the current finding of a longitudinal association between sleep problems and academic performance in school-aged children.
